# Entstehung und Verbreitung von Antibiotikaresistenzen in der Umwelt durch verstärkten anthropogenen Eintrag von Antibiotikarückständen und resistenten Erregern

**DOI:** 10.1007/s00103-026-04223-9

**Published:** 2026-04-13

**Authors:** Merit Maren Prüß, Sebastian Guenther

**Affiliations:** Pharmazeutische Biologie, Universität Greifswald, Friedrich-Ludwig-Jahn-Straße 17, 17489 Greifswald, Deutschland

**Keywords:** Antibiotikaresistenz, Antibiotikarückstände, Klimawandel, One Health, Umwelt, Antibiotic resistance, Antibiotic residues, Climate change, One Health, Environment

## Abstract

Die Anwendung von Antibiotika zur antimikrobiellen Therapie revolutionierte die medizinische Praxis. Jedoch hat die häufige und z. T. unsachgemäße Nutzung sowohl in der Human- als auch der Veterinärmedizin erhebliche Konsequenzen. Dieser Artikel gibt einen Überblick über Eintragspfade von Antibiotika in die Umwelt und die Auswirkungen.

Über Abwässer, Klärschlämme und Wirtschaftsdünger gelangen Antibiotika und ihre Abbauprodukte in verschiedene Umweltkompartimente wie Böden und Wasser, wo sie durch subinhibitorische Konzentrationen von Antibiotika die Entstehung und die Verbreitung antimikrobieller Resistenzen (AMR) fördern. Weltweit wurden in kommunalen Kläranlagen, Oberflächengewässern und landwirtschaftlichen Nutzflächen wiederholt Überschreitungen der ökotoxikologischen Schwellenwerte (*Predicted No Effect Concentration, PNEC*) festgestellt.

Subinhibitorische Umweltkonzentrationen verändern als Stressfaktor die Funktion mikrobieller Gemeinschaftsstrukturen und beeinträchtigen dadurch biogeochemische Kreisläufe. Es wurden bereits bidirektionale Wechselwirkungen zwischen Klimawandel und AMR postuliert, da zum einen durch veränderte mikrobielle Stoffwechselprozesse klimarelevante Rückkopplungen ausgelöst werden können und zum anderen Extremwetterereignisse wie Überschwemmungen die Verbreitung der AMR ungünstig beeinflussen. Die Resistenzproblematik erfordert daher einen integrativen, global koordinierten Ansatz im Sinne von One Health und Planetary Health.

## Abstract (en)

The use of antibiotics for antimicrobial therapy revolutionized medical practice. However, the frequent and sometimes inappropriate use of these drugs in both human and veterinary medicine has had significant consequences. This article provides an overview of the pathways by which antibiotics enter the environment and their impacts.

Through wastewater, sewage sludge, and agricultural fertilizers, antibiotics and their degradation products enter various environmental compartments such as soil and water, where they promote the emergence and spread of antimicrobial resistance (AMR) via subinhibitory concentrations of antibiotics. Worldwide, exceedances of ecotoxicological threshold values (predicted no effect concentrations; PNECs) have repeatedly been detected in municipal wastewater treatment plants, surface waters, and agricultural soils.

In addition, subinhibitory environmental concentrations act as stress factors that alter the structure and function of microbial communities, thereby disrupting biogeochemical cycles. Bidirectional interactions between climate change and AMR have already been postulated, since altered microbial metabolic processes may trigger climate-relevant feedback mechanisms, while extreme weather events such as floods can further facilitate the dissemination of AMR. The resistance problem therefore requires an integrated, globally coordinated approach in the sense of One Health and Planetary Health.

## Einleitung

Die Entdeckung und breite Anwendung von Antibiotika markierten einen Wendepunkt in der modernen Medizin. Ihr bestimmungsgemäßer und z. T. auch übermäßiger Einsatz in Human- und Veterinärmedizin sowie in der industriellen Tierhaltung hat jedoch erhebliche ökologische Folgen. Antibiotikarückstände gelangen in verschiedene Umweltkompartimente und erzeugen subinhibitorische Selektionsdrücke, die die Verbreitung antimikrobieller Resistenzen begünstigen. Zahlreiche Studien belegen zudem inzwischen eine weltweite Präsenz multiresistenter Erreger in natürlichen Ökosystemen [1, 2]. Diese Entwicklungen verstärken sich gegenseitig und stellen eine erhebliche Bedrohung für die Umwelt und die globale Gesundheit dar. Besonders besorgniserregend ist die Ausbreitung multiresistenter Erreger, die teils auch ohne direkten antibiotischen Selektionsdruck persistieren [2]. Seit Einführung der Antibiotika wurde eine zunehmende Kontamination von Umweltbereichen wie Oberflächen- und Grundwasser, Böden, Abwässern und Klärschlamm beobachtet. Diese fördert die Selektion resistenter Bakterien, die auch vom Menschen über die Umwelt aufgenommen werden können [1]. Neuere Forschung weist zudem darauf hin, dass der Eintrag von Antibiotika und resistenten Mikroorganismen globale Mikrobiome verändert und dadurch langfristig klimarelevante Prozesse beeinflussen könnte [3]. Verstärkt wird diese Entwicklung durch die Anwesenheit von Bioziden und Metallverbindungen aufgrund von Co-Selektionsmechanismen [4]. Im Folgenden werden die Eintragspfade der Antibiotika sowie deren Auswirkungen auf die Umwelt näher betrachtet.

## Wege des Eintrags von Antibiotika in die Umwelt

### Behandlung von Infektionskrankheiten.

**Abb. 1:**
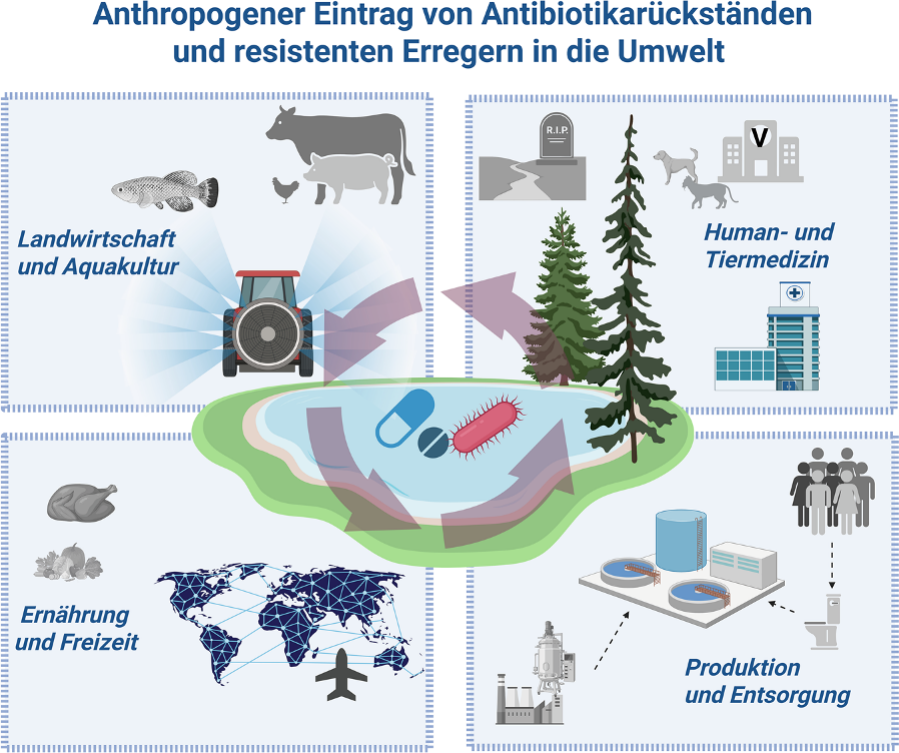
Anthropogener Eintrag von Antibiotikarückständen und resistenten Erregern in die Umwelt. Stark vereinfachte Darstellung. (Created with BioRender.com. Schultze, N. (2026) https://BioRopen acender.com/zr6qd2m)

Geringe Mengen antibiotisch wirksamer Substanzen werden von den natürlich in der Umwelt vorkommenden Mikroorganismen synthetisiert, um einen evolutionären Vorteil zu generieren. Daneben finden sich die menschengeschaffenen Eintragspfade, wie die Behandlung von Infektionskrankheiten mit Antibiotika (Abb. 1). Zwischen 40 % und 90 % der verabreichten Wirkstoffmenge werden in unveränderter, pharmakologisch aktiver Form über Fäzes und Urin ausgeschieden und anschließend in die Umwelt eingetragen [5]. Eine der bedeutendsten Eintragsquellen stellen kommunale und klinische Abwässer dar, die häufig unveränderte Antibiotika sowie deren Abbauprodukte enthalten.

​

In einer umfassenden Untersuchung von Rodriguez-Mozaz et al. [6], in der über 50 verschiedene Antibiotika in den Ablaufströmen kommunaler Kläranlagen aus 7 europäischen Ländern analysiert wurden, zeigten sich die höchsten Konzentrationen in Portugal, Spanien und Irland, während in Skandinavien, Deutschland und Zypern vergleichsweise niedrigere Werte gemessen wurden. Dennoch konnten auch in Deutschland Rückstände verschiedener Antibiotika flussabwärts von Kläranlageneinleitungen nachgewiesen werden – trotz der erheblichen Verdünnungseffekte, die im Gewässerlauf auftreten. Flussaufwärts wurden hingegen kaum Rückstände gefunden. Es wurden insbesondere Rückstände von Makrolid-Antibiotika wie Clarithromycin (bis zu 0,60 µg/L) sowie von Sulfamethoxazol (bis zu 0,40 µg/L) und Trimethoprim (bis zu 0,39 µg/L) nachgewiesen [7].

Der in Kläranlagen anfallende Klärschlamm stellt ebenfalls eine relevante Eintragsquelle dar, da Antibiotika darin nachweislich akkumulieren. Besonders hohe Konzentrationen wurden für Fluorchinolone ermittelt, insbesondere für Levofloxacin (212 µg/kg bis 2,49 mg/kg Trockenmasse [TM]) und Ciprofloxacin (576 µg/kg bis 6,75 mg/kg TM; [8]).

Aufgrund einer unvollständigen Abbauleistung in Kläranlagen können Antibiotika in die aquatische Umwelt freigesetzt werden, wobei die Entfernungseffizienz von dem jeweiligen Antibiotikum und der Substanzklasse abhängt [7]. Generell können Restkonzentrationen von Antibiotika bis zu etwa 120 μg/L in unbehandeltem Abwasser und bis zu etwa 8,0 μg/L in behandeltem Abwasser, das in Oberflächengewässer eingeleitet wird, erreichen [7]. Zusätzlich wurden in klinischen Abwässern höhere Restkonzentrationen bis in den 2‑stelligen μg/L-Bereich nachgewiesen [7, 9]. Abwassersysteme stellen daher eine potenzielle Punktquelle für das Auftreten und die Verbreitung von Antibiotika in der aquatischen Umwelt dar. In verschiedenen Studien wurden Makrolid-Antibiotika neben Sulfamethoxazol und Trimethoprim am häufigsten nachgewiesen [10].

Trotz dieser potenziellen Expositionsquelle zeigen jüngere Studien, dass nur niedrige Werte von Antibiotika in deutschen Badegewässern nachgewiesen werden konnten, was sich vor allem mit dem massiven Verdünnungseffekt erklären lässt [2, 11]. Dies bedeutet jedoch keine Entwarnung, da bekannt ist, dass auch sehr niedrige und umweltbedingt verdünnte Restkonzentrationen Antibiotikaresistenzen begünstigen [12, 13].

Die *Predicted No Effect Concentration* (vorhergesagte Konzentration ohne Wirkung, PNEC) beschreibt die höchste Konzentration eines Stoffes in der Umwelt, bei der keine schädlichen Effekte auf Organismen zu erwarten sind. Er dient somit als ökotoxikologischer Referenzwert für die Bewertung von Umweltrisiken. Untersuchungen zeigen, dass diese PNEC-Werte für verschiedene Antibiotika auch in Deutschland wiederholt überschritten wurden, insbesondere in den Abläufen kommunaler Kläranlagen sowie in Oberflächengewässern [14]. Dies weist darauf hin, dass lokale Konzentrationen von Antibiotikarückständen potenziell ökologische Auswirkungen haben und zur Selektion resistenter Mikroorganismen beitragen können. Der biologische Effekt dieser subinhibitorischen Konzentration ist also keinesfalls zu vernachlässigen.

### Industrielle Tierhaltung.

Auch die industrielle Tierhaltung stellt eine bedeutende Quelle für den Eintrag von Antibiotikarückständen in die Umwelt dar. Durch den umfangreichen Einsatz antimikrobieller Wirkstoffe in der Nutztierproduktion lassen sich Antibiotika regelmäßig in Wirtschaftsdüngern wie Gülle und Stallmist nachweisen. Zu den am häufigsten detektierten Substanzen zählen Amoxicillin, Enrofloxacin, Sulfadiazin und Tetracyclin [15]. Vor der Ausbringung von Gülle und Stallmist auf landwirtschaftliche Flächen erfolgt in der Regel keine Abwasserbehandlung. Aufgrund der teils hohen Persistenz bestimmter Antibiotika besteht somit ein erhebliches Risiko für eine Kontamination von Bodenporenwasser und Oberflächengewässern. Besonders Sulfadiazin, Tetracyclin und Trimethoprim wurden in erhöhten Konzentrationen im Bodenporenwasser nachgewiesen, was darauf hindeutet, dass diese Substanzen nach der Ausbringung über längere Zeiträume in der Umwelt verbleiben können.

In einer Metaanalyse von Frey et al. [16] wurden 57 in den vergangenen 2 Jahrzehnten veröffentlichte, begutachtete Studien ausgewertet, um einen umfassenden Überblick über das Vorkommen von Antibiotikarückständen in Mist, Gülle, Böden, Pflanzen und Gewässern zu erhalten. Die Analyse ergab, dass Schweinemist und Gülle aus europäischen Ländern häufig Rückstände von Fluorchinolonen, Sulfonamiden und Tetracyclinen aufwiesen. Zudem wurde festgestellt, dass der Einsatz von Antibiotika in der Tierhaltung zur Kontamination angrenzender Umweltkompartimente führt und in zahlreichen Fällen die von der EU vorgeschlagenen Grenzwerte für die maximale Bodenbelastung mit veterinärmedizinischen Antibiotika überschritten werden.

In Bodenproben wurden die höchsten Konzentrationen an Tetracyclinen und Sulfonamiden im Vereinigten Königreich (41 ± 18 µg/kg bzw. 300 ± 10 µg/kg) sowie für Fluorchinolone in Österreich (370 µg/kg) nachgewiesen. In aquatischen Proben lagen die Antibiotikagehalte in den meisten Ländern unter 2 µg/L; die höchsten Werte wurden für Fluorchinolone und Tetracycline in den USA (3 ± 0,7 µg/L bzw. 1,3 ± 0,6 µg/L) und für Sulfonamide in den USA und Deutschland (0,3 ± 0,8 µg/L bzw. 0,2 ± 2 µg/L) festgestellt. In Pflanzenproben wurde in China, Deutschland und Spanien eine Akkumulation von Tetracyclinen und Sulfonamiden über 50 µg/kg beobachtet. Diese Befunde verdeutlichen, dass Rückstände veterinärmedizinischer Antibiotika ein global verbreitetes Umweltproblem darstellen.

Die zunehmende Intensivierung der Aquakultur führt zu einer dichteren Tierhaltung und damit zu einem erhöhten Risiko für Krankheitsausbrüche. Zur Kontrolle und Behandlung infektiöser Erkrankungen kommen in der Aquakultur weltweit daher vermehrt antimikrobielle Wirkstoffe zum Einsatz [17], was wesentlich zur Umweltkontamination beiträgt, da Abwässer aus Aquakulturbetrieben direkt in die Umwelt eingeleitet werden. Im Jahr 2019 wurde der weltweite Verbrauch an Antibiotika in der Süßwasseraquakultur auf rund 12.252 t geschätzt, mit besonders hohen Einsatzmengen in der Asien-Pazifik-Region. Den größten Teil stellten Tetracycline dar, die etwa 70 % des gesamten Verbrauchs ausmachten. Zur Quantifizierung der durch die Aquakultur verursachten Süßwasserverschmutzung kann der sogenannte Graue Wasser-Fußabdruck herangezogen werden. Für die globale Aquakultur wird dieser auf 12.860 km^3^ pro Jahr geschätzt. Zum Vergleich beträgt der jährliche Abfluss des Amazonas, des volumenstärksten Flusses der Erde, rund 6595 km^3^. Diese Größenordnung verdeutlicht den erheblichen Beitrag der Aquakultur zum weltweiten Eintrag antimikrobieller Substanzen in aquatische Systeme [17].

### Pharmazeutische Industrie.

Auch die pharmazeutische Industrie trägt wesentlich zur Umweltbelastung durch Antibiotika bei. Produktionsstätten, insbesondere in Ländern mit unzureichenden Umweltauflagen, können erhebliche Mengen an Wirkstoffen in die Umwelt freisetzen. Eine Studie aus Indien dokumentierte extrem hohe Antibiotikakonzentrationen in Fließgewässern in der Nähe pharmazeutischer Produktionsanlagen [18].

Eine aktuelle Untersuchung der AOK Baden-Württemberg in Kooperation mit dem Umweltbundesamt (UBA) und dem Institut für Wasserforschung (IWW) bestätigte vergleichbare Befunde aber auch für Europa. In der Nähe von Produktionsstätten für Antibiotika wurden signifikant höhere Wirkstoffkonzentrationen in Gewässern gemessen als in weniger belasteten Regionen [19]. An rund 40 % der untersuchten Standorte überschritten die Konzentrationen im Abwasser oder in der angrenzenden Umwelt die vertraglich festgelegten Grenzwerte (PNEC) teils um ein Vielfaches. So lag die Ciprofloxacin-Konzentration im Abwasser um bis zu 11.000 % und die Azithromycin-Konzentration im Wasser um 1.600.000 % über den jeweiligen Referenzwerten (PNECs). Die Belastung war nicht auf Produktionsstandorte beschränkt, sondern erstreckte sich auch auf angrenzende Ökosysteme, was auf eine potenzielle Förderung von Antibiotikaresistenzen hinweist. Die Ergebnisse der Pilotstudie verdeutlichen einen dringenden Handlungsbedarf zur Minderung industrieller Emissionen und zur Stärkung ökologischer Nachhaltigkeitskriterien in der Antibiotikaproduktion, einschließlich regulatorischer Maßnahmen auf europäischer Ebene.

## Einfluss von Antibiotikarückständen auf die Resistenzentwicklung

Hohe Konzentrationen antimikrobieller Substanzen fördern bekanntermaßen die Selektion resistenter Bakterienpopulationen. Neuere Untersuchungen zeigen jedoch, dass auch subinhibitorische, umweltbedingt verdünnte Konzentrationen die Resistenzentwicklung begünstigen können [12, 13]. Kläranlagen gelten dabei als zentrale Hotspots [20], da sie durch den Eintrag von Antibiotika über Abwässer zur Anreicherung resistenter Bakterien beitragen. Insbesondere der Eintrag über kommunale und tierhaltungsbedingte Abwässer stellt ein wesentliches Risiko für die Resistenzbildung in agrarischen Ökosystemen dar. Langzeituntersuchungen belegen, dass Böden, die wiederholt mit antibiotikahaltiger Gülle behandelt wurden, signifikant höhere Konzentrationen an Resistenzgenen aufweisen als unbehandelte Vergleichsflächen [21, 22].

Resistenzen entstehen durch Mutationen oder horizontalen Gentransfer und auch bei geringen Rückstandskonzentrationen kann der bestehende Selektionsdruck das Überleben resistenter Stämme gegenüber sensiblen Populationen fördern und somit deren Anteil in der Umwelt erhöhen. Zusätzlich induzieren subinhibitorische Antibiotikakonzentrationen Veränderungen der bakteriellen Genexpression, insbesondere im Rahmen von Stressantworten [20]. Die Anwesenheit von Bioziden sowie Metallverbindungen verstärkt die Stressantwort und führt durch die Co-Lokalisation der entsprechenden Resistenzgene auf den Plasmiden zu einer Co-Selektion [4, 23]. Die Folge ist eine Begünstigung der Resistenzentwicklung auch gegen Antibiotika.

Die Verbreitung resistenter Bakterien erfolgt über Wasserläufe, Böden und die Nahrungskette. Menschen und Tiere tragen zur Dissemination bei, indem sie resistente Mikroorganismen ausscheiden (Abb. 1). Auch die Nutzung unzureichend behandelter Abwässer zur landwirtschaftlichen Bewässerung stellt einen relevanten Eintragspfad dar. Eine systematische Auswertung von Treskova et al. [24] zu Antibiotikaresistenzen in Umweltkompartimenten Deutschlands, Österreichs und der Schweiz, basierend auf 400 Datensätzen, verdeutlicht das Ausmaß: Resistenzen gegen sämtliche WHO-kritischen Wirkstoffklassen wurden in Oberflächen- und Badegewässern, in Abwässern sowie in Proben aus Tierhaltung und Sedimenten nachgewiesen. Besonders häufig wurden Resistenzgene gegen Fluorchinolone, Cephalosporine, Glykopeptide und Colistin identifiziert. Der Verzehr von rohen Pflanzen, welche mit unzureichend aufgearbeitetem Wasser bewässert werden, sorgt überdies für eine verstärkte Exposition der Verbraucher gegenüber übertragbaren Resistenzgenen [25].

Ein bisher wenig beachteter Aspekt betrifft das sogenannte Thanatoresistom, also das Resistom nach dem Tod. Untersuchungen weisen darauf hin, dass resistente Mikroorganismen auch postmortal persistieren und sich mangels immunologischer Kontrolle weiter ausbreiten können. Während des Zersetzungsprozesses können sie über Organismen wie Insektenlarven, Würmer oder Pilze, die an der Verwesung beteiligt sind, in die Nahrungskette gelangen. Zudem wird Sickerwasser von Friedhöfen in der Regel nicht behandelt, sodass Resistenzgene und verbliebene Antibiotikarückstände ungehindert in den Wasserkreislauf gelangen können [26].

Besonders relevant sind zudem multiresistente Erreger, die sowohl im klinischen als auch im Umweltkontext eine zunehmende Bedrohung darstellen. High-risk-clonal Lineages (z. B. E. coli ST131, ST648, ST410) sind gegen mehrere Wirkstoffklassen resistent und wurden weltweit in Wildtieren nachgewiesen [27–29]. Dies verdeutlicht, dass klinisch relevante Stämme auch außerhalb des hohen Selektionsdrucks in Klinik- oder Tierhaltungssystemen überleben und in Umweltkompartimenten persistieren können. Neben dieser Sentinel- bzw. Bioindikatorfunktion wird die Rolle von Wildtieren zunehmend als bedeutender Vektor in der Resistenzverbreitung diskutiert. Synanthrope und migratorische Vogelarten spielen hierbei eine Schlüsselrolle, da sie resistente Bakterien über weite Distanzen, teils kontinentübergreifend, verbreiten [30, 31]. Die globale Dissemination dieser Erreger unterstreicht die Notwendigkeit international koordinierter Überwachungs- und Kontrollstrategien [32].

## Erweiterte Umweltfolgen durch den Eintrag von Antibiotika

Die Folgen des Eintrags von Antibiotika in die Umwelt sind komplex und gehen weit über die Resistenzproblematik bei Pathogenen hinaus [33]. Antibiotika sind explizit so konzipiert, dass sie einen Effekt auf Mikroorganismen haben, und obwohl bakterizide Konzentrationen selten außerhalb therapeutischer Anwendungen auftreten, können auch subinhibitorische Konzentrationen von Antibiotika schädliche Effekte auf natürliche mikrobielle Gemeinschaften haben, da sie eine Hemmung vieler Gruppen von Mikroorganismen hervorrufen können, die an Schlüsselökosystemfunktionen beteiligt sind.

Mikrobielle Biodiversität hat eine funktionelle Bedeutung bei der Aufrechterhaltung biologischer Prozesse in Wasser und Boden; tatsächlich werden die meisten biogeochemischen Kreisläufe ausschließlich durch Mikroorganismen ermöglicht [33]. Antibiotika können als ökologischer Faktor in der Umwelt wirken und Änderungen in der Struktur natürlicher bakterieller Gemeinschaften bewirken. Die Auswirkungen können sogar bei Nichtzielorganismen wie Pilzen oder Pflanzen mit wichtigen ökologischen Funktionen gefunden werden [33]. Die Präsenz von Antibiotika verursacht eine Reduktion der mikrobiellen Biodiversität. Darüber hinaus können sie das Wachstum und die Enzymaktivitäten von bakteriellen Gemeinschaften beeinflussen und letztendlich ökologische Funktionen wie Biomasseproduktion und Nährstofftransformation beeinträchtigen, was zu einem Verlust funktionaler Stabilität führt. Der selektive Effekt von Antibiotika auf verschiedene mikrobielle Gruppen ändert die relative Häufigkeit mikrobieller Arten und stört Interaktionen zwischen verschiedenen Arten. Diese Effekte hängen von den beteiligten mikrobiellen Gruppen, Umweltmerkmalen und von Antibiotikakonzentrationen ab. Zu den Auswirkungen von Antibiotika auf ökologische Funktionen gehören Änderungen in der Stickstofftransformation, Methanbildung, Schwefelreduktion und Nährstoffkreisläufen [33].

Direkte nachteilige Effekte auf andere Gruppen von Nichtzielorganismen sind beispielsweise eine starke Vermehrung von Cyanobakterien bei Umweltkonzentrationen von etwa 300 ng/L der Wirkstoffe Sulfamethoxazol, Tetrazyklin und Ciprofloxacin [34]. Bei höheren Konzentrationen hingegen wurden wachstumshemmende Effekte auf Cyanobakterien sowie auf eukaryotische Algen beobachtet [35]. Auch für höhere Pflanzen wurde eine durch das Vorhandensein von Antibiotika hervorgerufene Reduktion der Keimungsrate nachgewiesen [36]. Die subtile, aber tiefgreifende Ausweitung des Antibiotikaeinflusses verdeutlicht, dass die Problematik längst über den Rahmen des One-Health-Ansatzes hinausreicht und bereits die übergeordneten Zusammenhänge der *Planetary Health* betrifft.

## Einfluss durch und auf den Klimawandel

Der Zusammenhang zwischen Klimawandel und Antibiotikaresistenz stellt ein vergleichsweise neues, jedoch zunehmend relevantes Forschungsfeld dar. Zahlreiche Befunde deuten darauf hin, dass klimatische Veränderungen die Verbreitung multiresistenter Mikroorganismen begünstigen. So konnte gezeigt werden, dass salz- und thermostabile Bakterien häufig zugleich Resistenzen gegenüber antimikrobiellen Substanzen aufweisen [37]. Steigende Temperaturen und veränderte Salzgehalte in den Ozeanen fördern die Ausbreitung solcher Mikroorganismen. Darüber hinaus begünstigen Extremwetterereignisse, wie Überschwemmungen, die Verschleppung multiresistenter Erreger in neue ökologische Nischen, darunter Flüsse, Seen und landwirtschaftlich genutzte Flächen [37].

Die Wechselwirkungen zwischen Klimawandel und antimikrobieller Resistenz (AMR) sind potenziell aber bidirektional. In einem jüngst veröffentlichten Review, der die Schnittstelle beider Phänomene untersucht, wurde die Hypothese aufgestellt, dass steigende Temperaturen und die Ausbreitung resistenter Mikroorganismen synergistisch auf Bodenmikroben wirken und deren Kohlenstoffnutzungseffizienz (CUE) verringern [3]. Dadurch wird weniger Kohlenstoff in mikrobielle Biomasse eingebaut und vermehrt als CO_2_ freigesetzt, was die Kohlenstoffspeicherung in Böden mindert und klimawirksame Rückkopplungseffekte verstärkt. Erwärmungsbedingte Steigerungen mikrobieller Stoffwechselaktivität und AMR-bedingte energetische Belastungen durch Resistenzmechanismen könnten somit gemeinsam zur Destabilisierung des globalen Boden-Kohlenstoffkreislaufs beitragen. Besonders gefährdet sind arktische Permafrostböden, tropische Ökosysteme und agrarisch genutzte Flächen, in denen Antibiotikaeinträge und Temperaturanstiege zusammenwirken. Die Autoren beschreiben einen potenziellen Rückkopplungseffekt, bei dem AMR und Klimawandel sich gegenseitig verstärken und sowohl ökologische als auch gesundheitliche Risiken erhöhen [3].

## Fazit

Die zunehmende Verbreitung von Antibiotikarückstanden und antimikrobiellen Resistenzen in der Umwelt zeigt, dass es sich nicht mehr um ein ausschließlich medizinisches, sondern um ein globales Umwelt- und Gesundheitsproblem handelt. Der fortgesetzte Eintrag von Antibiotika und resistenten Mikroorganismen verändert mikrobielle Gemeinschaften und beeinträchtigt zentrale ökologische Prozesse. Dadurch entstehen Risiken für die menschliche Gesundheit, etwa durch Kontamination von Wasser, Nahrungsmitteln und Böden. Damit breitet sich die Resistenzproblematik zunehmend aus der Human- und Veterinärmedizin in die Umwelt aus. Viele Nosokomialkeime mit ausgeprägten Resistenzmechanismen und der Fähigkeit, Biofilme auszubilden, sind obendrein auch eng mit Pflanzen assoziiert. Darunter fallen beispielsweise Infektionen mit Enterobacter und Klebsiella. Zugleich bestehen potenzielle Wechselwirkungen zwischen Klimawandel und Resistenzentwicklung. Erwärmung, veränderte Niederschlagsmuster und Extremereignisse fördern die Verbreitung resistenter Mikroorganismen und verändern mikrobiologische Ökosysteme. Umgekehrt könnten Antibiotikarückstände und AMR klimarelevante Prozesse, insbesondere den Kohlenstoffkreislauf, beeinflussen. Es entsteht ein potenziell bidirektionaler Wirkungszusammenhang, in dem Umweltveränderungen und Resistenzbildung sich gegenseitig verstärken.

Die Bewältigung dieser Herausforderung erfordert integrierte, interdisziplinäre Strategien im Sinne von One Health und Planetary Health. Neben einer Reduktion des Antibiotikaverbrauchs sind verbesserte Abwasserbehandlung, strengere Emissionsgrenzwerte in der Arzneimittelproduktion sowie nachhaltige landwirtschaftliche Praktiken entscheidend. Die Resistenzkrise verdeutlicht die Grenzen menschlicher Eingriffe in mikrobiologische Systeme. Gesundheit von Mensch, Tier und Umwelt muss als untrennbar verbundenes Ganzes verstanden werden. Nur durch einen integrierten, global koordinierten Ansatz lässt sich die Ausbreitung antimikrobieller Resistenzen eindämmen und die ökologische Grundlage künftiger Generationen bewahren.
